# In-situ scalable manufacturing of Epstein–Barr virus-specific T-cells using bioreactor with an expandable culture area (BECA)

**DOI:** 10.1038/s41598-022-11015-z

**Published:** 2022-04-29

**Authors:** Sixun Chen, Ahmad Amirul Bin Abdul Rahim, Who-Whong Wang, Rachael Cheong, Akshaya V. Prabhu, Jerome Zu Yao Tan, May Win Naing, Han Chong Toh, Dan Liu

**Affiliations:** 1 Biomanufacturing Technology, Bioprocessing Technology Institute, Agency for Science, Technology and Research (A*STAR), 20 Biopolis Way, Singapore, 138668 Singapore; 2 Division of Medical Oncology, National Cancer Centre Singapore, 11 Hospital Cres, Singapore, 169610 Singapore; 3 Singapore Institute of Manufacturing Technology (SIMTech), A*STAR, 2 Fusionopolis Way, Singapore, 138634 Singapore; 4 Interdisciplinary Graduate Programme, School of Chemical and Biomedical Engineering, Nanyang Technological University, Singapore, Singapore

**Keywords:** Cell therapies, Biological techniques, Engineering

## Abstract

The ex-vivo expansion of antigen-specific T-cells for adoptive T-cell immunotherapy requires active interaction between T-cells and antigen-presenting cells therefore culture density and environment become important variables to control. Maintenance of culture density in a static environment is traditionally performed by the expansion of the culture area through splitting of culture from a single vessel into multiple vessels—a highly laborious process. This study aims to validate the use and efficacy of a novel bioreactor, bioreactor with an expandable culture area—dual chamber (BECA-D), that was designed and developed with a cell chamber with expandable culture area (12–108 cm^2^) and a separate media chamber to allow for in-situ scaling of culture with maintenance of optimum culture density and improved nutrient and gas exchange while minimizing disturbance to the culture. The performance of BECA-D in the culture of Epstein–Barr virus-specific T-cells (EBVSTs) was compared to the 24-well plate. BECA-D had 0.9–9.7 times the average culture yield of the 24-well plates across 5 donor sets. BECA-D was able to maintain the culture environment with relatively stable glucose and lactate levels as the culture expanded. This study concludes that BECA-D can support the culture of ex-vivo EBVSTs with lower manufacturing labour and time requirements compared to the use of the 24-well plate. BECA-D and its adaptation into a closed system with an automated platform (currently being developed) provides cell therapy manufacturers and developers with a closed scale-out solution to producing adoptive cell therapy for clinical use.

## Introduction

Adoptive T-cell immunotherapy has been the focus of development for new treatment modalities in the clinic. The potential of T-cell therapies is evidenced by the success of Chimeric Antigen Receptor T-cell (CAR-T) therapy in refractory hematologic malignancies^1–3^. Several positive T-cell therapy clinical trials have led to multiple US Food and Drug Administration (FDA) approvals from 2017 to 2021^4^. In addition to CAR-T therapies, adoptive antigen-specific T-cell therapies such as Epstein–Barr virus-specific T-cells (EBVSTs) for EBV-associated malignancies have also shown positive results in clinical trials^5^. Despite their promising efficacy, T-cell therapies generally require costly and time-consuming manufacturing processes. In particular, starting material variation and sensitivity of T-cells can lead to manufacturing failure when insufficient cells are harvested at the end of culture^6,7^. The limitations in manufacturing severely restricts the widespread use of T-cell therapies.

The manufacturing process of antigen-specific T-cells involves ex-vivo co-culture of donor T-cells with antigen-presenting cells (APCs). Optimal cell–cell interaction between the two populations is crucial in achieving activation and expansion of the T-cells^8^. Optimal cell–cell interactions can be achieved with the control of culture density. Lab-scale culture and expansion of antigen-specific T-cells performed solely in 24-well plates maintains the cell density and cell–cell interactions in a familiar constrained culture environment. However, processes performed in 24-well plates are not amenable to scaling for clinical manufacturing as the handling gets increasingly laborious with culture expansion and increase in number of patients. The scaling and expansion of suspension cells are often performed in commercially-available bioreactors which have been designed to control culture density by adjusting the volume of media in the suspended culture. However, T-cells are semi-adherent in nature indicating that their culture density is influenced by culture surface area (cells/cm^2^) rather than culture volume (cells/mL), making these bioreactors unsuitable for T-cell culture. These bioreactors also utilize agitation such as stirring, shaking, and rocking to introduce gas and nutrient exchange, putting cells in constant motion which does not confer an ideal environment for sustaining cell–cell interactions. Sustained interactions between co-culture of T-cells and APCs are key to the production of T-cells with activity and specificity towards these antigens^8^. Currently, there is no stand-alone bioreactor in the commercial market that incorporates culture surface area as a critical parameter to control.

Taking into considerations the benefits and limitations of the 24-well plate and commercially-available bioreactors, we have designed a Bioreactor with Expandable Culture Area (BECA) suitable for the expansion of antigen-specific T-cells^9^. BECA allows for the expansion of culture surface area in the same vessel (in situ) as the cells proliferate, maintaining culture density while keeping cells in static culture condition. We have developed two variations of BECA, with dual chamber (BECA-D) and single chamber (BECA-S). Apart from the culture of antigen-specific T-cells, the suite of BECA bioreactors can be adapted for a wide range of culture protocols. BECA-D, consisting of an expandable cell chamber and an attached media chamber, would be suitable for culture protocols that require co-culture (as observed for antigen-specific T-cells and APCs in this study) or utilize media change as feeding strategy. BECA-S, consisting of an expandable cell chamber, would be more suitable for use in culture protocols that utilize media-top up as feeding strategy.

We have previously shown that BECA-D was able to support the growth of Jurkat cell line and T-cells activated by CD3/CD28 Dynabeads^10^. In this study, BECA-D was evaluated for its performance in the expansion of healthy donor-derived EBVSTs activated by co-culturing with autologous APCs. BECA-D’s performance was compared with that of the 24-well plate with the aim to match yield (cell population), viability, phenotype and functionality of the culture in 24-well plate while reducing handling steps, especially open cultureware manipulations.

## Results

### BECA-D design for the optimal expansion of semi-adherent cells

**Figure 1:**
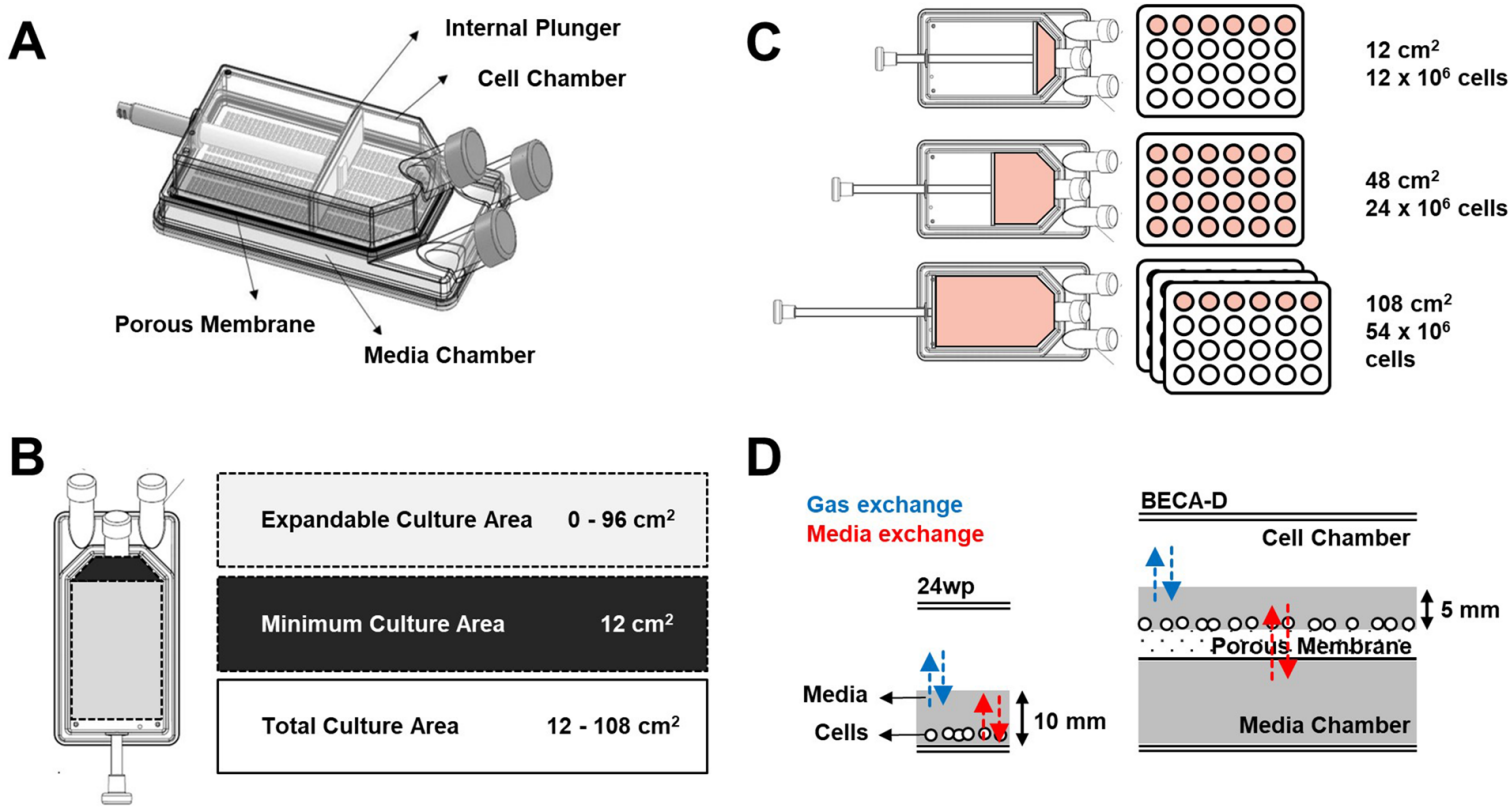
Design of bioreactor with an expandable culture area—dual chamber (BECA-D). (**A**) Schematic of BECA-D. (**B**) Culture surface area that can be expanded by manipulating the internal plunger to achieve total culture area of 12–108 cm^2^. (**C**) Comparison of culture expansion in BECA-D and 24-well plate. (**D**) Comparison of media and gas exchange in a 24-well plate and BECA-D. Porous membrane and dual chamber in BECA-D enable bulk nutrient supply from medium chamber and efficient gas exchange in cell chamber.

The patent pending bioreactor (WO2018/182533 A1), BECA-D, comprises of a cell chamber on top of a media chamber, a porous membrane between the cell chamber and the media chamber, and an internal plunger which can be used to adjust the culture surface area of the cell chamber (Fig. 1A). The design emphasizes features that are beneficial to culture of T-cells and semi-adherent cells: maintenance of culture density^11^, provision of bulk nutrient^12^, reduction of disturbance to the cells^8^, and provision of adequate oxygen supply^13,14^.

The incorporation of an internal plunger in the cell chamber allows for the straightforward adjustment of culture density (cells/cm^2^) in situ. The plunger separates the chamber into two sections, the front and the back. Cells are cultured in the front section where the culture surface area can be expanded by pulling the plunger towards the back of the bioreactor. This increases the cell culture area and lowers the culture density (cells/cm^2^) to effectively maintain an optimal culture density as the cell population increases during culture. The minimum culture area for BECA is 12 cm^2^ and it is expandable up to total culture area of 108 cm^2^ (Fig. 1B). This feature allows the culture to be expanded within the same vessel. In comparison, culture expansion in standard culture vessels would require additional handling—pooling cells from the original vessel and seeding the expanded culture population into multiple new vessels of the same culture area, or vessels of a larger culture area (Fig. 1C).

The provision of bulk nutrient, and reduction of disturbance to the cells are achieved through the design of a separate but connected bulk media source in BECA-D—the media chamber. The media chamber and porous membrane enable nutrients (e.g. glucose) from the bulk media to be continuously supplied to the cells in the cell chamber (Fig. 1D). Metabolite wastes (e.g. lactate) would also be continuously diluted out from the cell chamber into the media chamber. By slowing the depletion of nutrients and accumulation of waste, this feature couple simplify culture processes by allowing for fewer media changes. The separate chambers design aims at minimizing change and turbulence to the immediate surroundings of the culture as any media change or top-up of additional reagents (e.g. cytokines) can be done through the media chamber without affecting the immediate culture environment in the cell chamber.

In standard culture vessels, nutrient provision and accessibility to atmospheric oxygen have to be carefully balanced as increasing the volume of media in the culture vessel would increase the media height and thus compromising on the efficiency of oxygen delivery from the atmosphere to the cells. In BECA-D, the roles of nutrient provision and accessibility to atmospheric oxygen are clearly separated between the Media and cell chamber respectively. As cells in the cell chamber are supplied with media from the separate media chamber, it is thus feasible to maintain a low media height in the cell chamber allowing for higher efficiency of oxygen delivery from the headspace of the vessel to the cells. In comparison to the 24-well plate, the design of BECA-D’s Media and cell chamber improves both nutrient provision and oxygen delivery to the culture, providing optimal conditions for growth (Fig. 1D).

### EBVST culture achieved highest yield with direct seeding into BECA-D at a higher seeding density

The protocol for EBVST culture was previously optimized for the 24-well plate (data not shown). Briefly, PBMCs are seeded at a density of 1.0 × 10^6^ cells/cm^2^ for 1st activation and the culture is maintained at 0.5 × 10^6^ cells/cm^2^ for subsequent activations. To optimize the protocol for EBVST culture for BECA-D, we tested two hypotheses. First, we hypothesized that PBMCs conditioned for culture in 24-well plate in a smaller media volume for the 1st activation and transferred to BECA-D for subsequent activations might experience enhanced proliferation rates compared to PBMCs directly seeded into BECA-D. The smaller media volume in the 24-well plate would provide a higher concentration of paracrine and autocrine signalling molecules secreted by activated T-cells which could be crucial for its proliferation. Second, we hypothesized that seeding density of 24-well plate conditioned PBMCs could affect culture proliferation in BECA.

**Figure 2:**
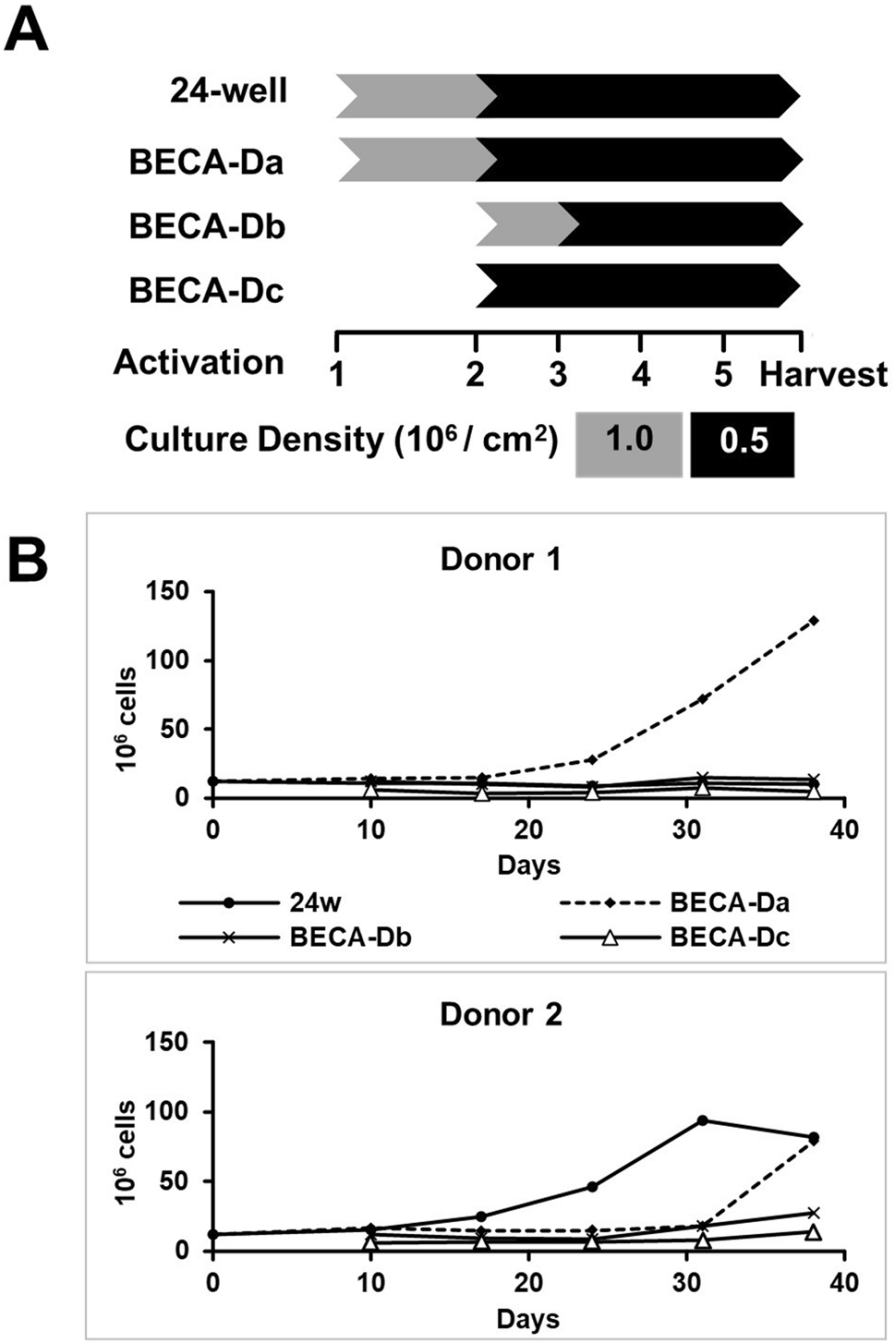
Expansion of EBVST in 24-well plate (24w), BECA-Da, BECA-Db, BECA-Dc. (**A**) Experimental scheme. Cultures in 24-well plate (24w), BECA-Da and BECA-Db were seeded at 1.0 × 10^6^ cells/cm^2^ and maintained in 0.5 × 10^6^ cells/cm^2^ while culture in BECA-Dc was seeded and maintained at 0.5 × 10^6^ cells/cm^2^. Culture in 24-well plate and BECA-Da was initiated on 1st activation while cultures in BECA-Db and BECA-Dc were initiated in 24-well plate on 1st activation then transferred to BECA-D on 2nd activation. (**B**) Plot of cell population numbers counted for each vessel at the end of every activation for Donor 1 and Donor 2.

An experiment was set up to test these hypotheses with three different setups for BECA-D (BECA-Da, BECA-Db and BECA-Dc respectively) with varying seeding timepoints into BECA-D and seeding densities. BECA-Da was seeded on Day 0 (1st activation) at 1.0 × 10^6^ cells/cm^2^ and maintained at 0.5 × 10^6^ cells/cm^2^, BECA-Db was seeded on Day 10 (2nd activation) from a culture conditioned in 24-well plate at 1.0 × 10^6^ cells/cm^2^ and maintained at 0.5 × 10^6^ cells/cm^2^ and BECA-Dc was seeded on Day 10 (2nd activation) from a culture conditioned in 24-well plate at 0.5 × 10^6^ cells/cm^2^ and maintained at 0.5 × 10^6^ cells/cm^2^. The experiment was conducted twice with PBMCs obtained from two separate healthy donors, Donor 1 and Donor 2, over the course of 5 rounds of activations with a culture in a 24-well plate as the control (Fig. 2A).

Comparing among the different BECA-D protocols, BECA-Da (Donor 1 and Donor 2) achieved higher yield (129.1 and 79.1 × 10^6^ cells) than BECA-Db (13.8 and 27.6 × 10^6^ cells) and BECA-Dc (4.9 and 13.7 × 10^6^ cells), suggesting that the conditioning of PBMCs in 24-well plate for the 1st activation did not confer any benefit to culture proliferation (Fig. 2B, Table 1). At the end of 5th activation, the culture in BECA-Da was observed to be in exponential phase of growth indicating the potential to further expand the cell population if the culture were to continue. Taken together, BECA-Da conferred the optimal culture conditions for the expansion of EBVSTs, achieving yield exceeding or close to the 24-well plate. Based on these results, the BECA-Da’s expansion protocol was utilized in subsequent experiments in this study.

**Table 1:** Expansion of EBVST in 24-well plate (24w) and BECA-D.

	Vessel	Donor 1	Donor 2	Donor 3	Donor 4	Donor 5	Paired T-test
**Yield/viability**							
Cell population 10^6^ cells (% viability)	24w	10.3 (90%)	81.6 (78%)	28.8 (69%)	59.5 (85%)	87.4 (94%)	p = 0.13 *n.s.*
	BECA-D	129.1 (95%)	79.1 (91%)	47.7 (81%)	160.0 (90%)	91.9 (91%)	
**Phenotype**							
% CD3+	24w	93.5	96.8	95.0	95.1	93.7	p = 0.54 *n.s.*
	BECA-D	96.9	95.4	95.6	96.0	93.0	
% CD19+	24w	< 0.1	< 0.1	< 0.1	< 0.1	< 0.1	p = 0.45 *n.s.*
	BECA-D	< 0.1	< 0.1	< 0.1	< 0.1	< 0.1	
% CD56+	24w	20.6	1.5	0.6	0.5	0.6	p = 0.78 *n.s.*
	BECA-D	7.6	5.3	2.8	3.1	0.3	
% CD3+/CD4+	24w	8.8	15.9	5.8	55.8	6.8	p = 0.31 *n.s.*
	BECA-D	8.1	12.8	8.8	35.7	5.4	
% CD3+/CD8+	24w	81.0	56.7	87.6	41.6	82.4	p = 0.06 *n.s.*
	BECA-D	87.8	59.0	87.1	53.9	89.4	
**Functionality**							
ELISpot (SFU/10^6^ cells)	24w	47	1381	558	239	616	p = 0.24 *n.s.*
	BECA-D	87	1394	557	1002	791	
Cytotoxicity E:T ratio, 20:1 (% specific killing)	24w	39.7	20.7	34.3	35.8	55.0	p = 0.65 *n.s.*
	BECA-D	45.3	31.2	48.0	14.7	61.6	

Summary of results (yield, viability, phenotype and functionality) of the culture in 24-well plate and BECA-D from five different donor runs. Paired T-test was performed for each set of results to determine significant differences between them.

### EBVST culture from 4 out of 5 donors achieved higher yield in BECA-D compared to 24-well plate

**Figure 3:**
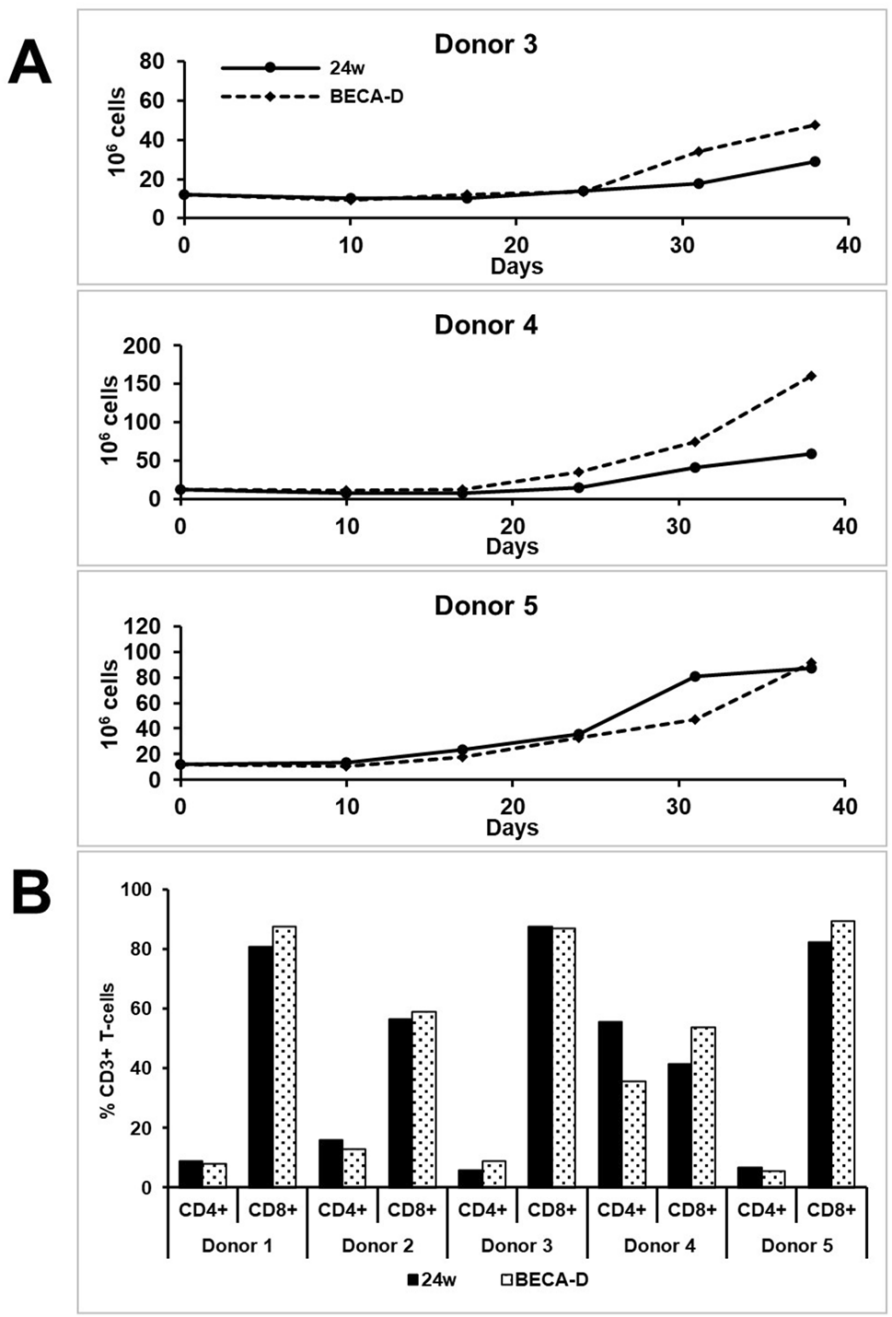
Expansion of EBVST in 24-well plate (24w) and BECA-D. (**A**) Plot of cell population numbers counted for 24-well plate (24w) and BECA-D at the end of every activation for Donor 3, Donor 4 and Donor 5. (**B**) Percentage population of helper (CD4+) and cytotoxic (CD8+) T-cells under the subset of T-cells (CD3+) cells at the end of 5th activation for Donors 1–5.

We repeated BECA-Da’s expansion protocol for PBMCs from three separate healthy donors (Donor 3, Donor 4 and Donor 5). The results were compared with respective donors’ cultures concurrently performed in 24-well plates. In all three donors, BECA-D achieved high viability and higher yield than 24-well plate (Fig. 3A, Table 1). Combined results from the five experiments (Donor 1–5) are presented in Table 1. Paired Student’s two-tailed T-test was performed to statistically determine the difference between 24-well plate and BECA-D’s performance in the culture of EBVSTs from each donor. Overall, there was no significant difference observed between the performance of 24-well plate and BECA-D in terms of cell population and viability across five donors (Table 1).

The phenotype and functionality of the cultures were also assessed to determine if harvested cells at the end of expansion process achieved the desired quality attributes of EBVST. Both 24-well plate and BECA-D achieved > 90% of CD3+ T-cells and < 0.1% of CD19+ B-cells (Table 1, Supplementary Fig. 1A). BECA-D had consistently low population percentages of CD56+ NK cells (< 10%) compared to 24-well plate. Inter-donor variability was observed to be higher when assessing the ratio of CD4+ helper T-cells and CD8+ cytotoxic T-cells. However, within each donor, the ratio in BECA-D were observed to match well with no significant difference from the ratio in 24-well plate (Fig. 3B, Table 1).

The functional potency of the expanded cells was assessed through two assays: an ELISpot assay to quantify IFN-γ secretion upon exposure to EBV antigens (LMP1, LMP2 and EBNA1) and a killing assay against autologous APCs. EBVST from both BECA-D and 24-well plate were observed to have robust secretion of IFN-γ upon exposure to EBV antigens, with four donor samples achieving similar total Spot Forming Units (SFU) per 10^6^ cells in BECA-D and 24-well plate (Table 1). EBVST from five BECA-Ds and four 24-well plates were also able to kill autologous APCs achieving ≥ 20% specific killing rate at 20:1 of effector:target (E:T) ratio, indicating functionally cytolytic T-cells (Table 1, Supplementary Fig. 1B). Results from the functional assays were observed to have no significance difference between BECA-D and 24-well plate (Table 1).

### Bioreactor environment maintained uniform glucose level in culture

One of the key design features of BECA-D is the dual chamber design consisting of the media chamber which holds excess media providing a bulk nutrient supply for the cell chamber. We hypothesized that the excess media would provide a buffer against depletion of nutrients such as glucose and production of wastes such as lactate throughout the culture process. In the experiments performed for Donor 3, 4 and 5, we measured the glucose and lactate concentrations in the media of both 24-well plate and BECA-D (cell chamber (CC) and media chamber (MC)) at the end of each activation round prior to media change. Glucose levels are reported as a percentage with respect to levels in fresh media to track its depletion through the culture and lactate levels are reported in concentration in the media (mM) to track its accumulation through the culture (lactate concentration was measured to be 0 mM in fresh media).

**Figure 4:**
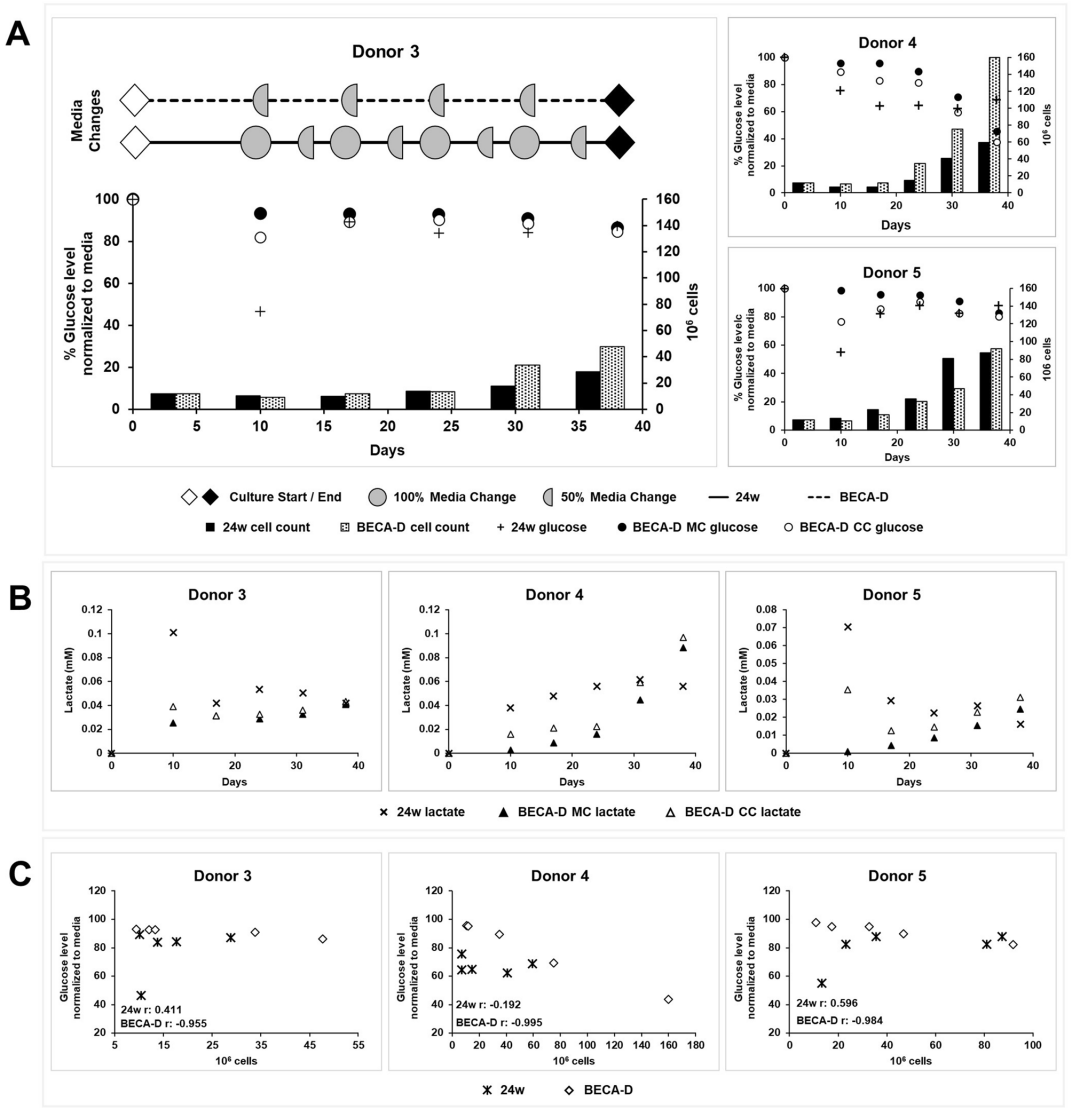
Glucose and lactate levels in 24-well plate (24w) and BECA-D during EBVST culture. (**A**) Normalized glucose level (markers) in media collected and cell count (bar graph) in 24-well plate (24w) and BECA-D at the end of each activation. *MC* media chamber, *CC* cell chamber. Representative media change frequencies in 24-well plate and BECA-D are shown in Donor 3 graph. (**B**) Lactate concentration in media collected in 24-well plate (24w) and BECA-D at the end of each activation. (**C**) Plot of cell count against matched normalized glucose level in 24-well plate (24w) and BECA-D starting from 2nd activation, r value was calculated using Pearson correlation coefficient.

There was an observable decrease in glucose and increase in lactate levels in the culture media upon 1st activation in both 24-well plate and BECA-D cell chamber (Fig. 4A,B). This indicates the increase in glucose consumption and lactate production upon the 1st round of T-cell activation. In each round of activation, 24-well plate had a 100% media change on day 0 and a 50% media change on the day 4 while BECA-D had a 50% media change on day 0 (Fig. 4A). The media changes refreshed the glucose level in the vessels—24-well plate to 100% on day 0 and 50–100% on day 4, and BECA-D to 50–100% on day 0.

In Donor 3 and 5, there was a gradual decrease in glucose and increase in lactate levels in BECA-D throughout the process but in Donor 4, a drastic decrease in glucose and increase in lactate levels was observed in BECA-D from Day 24 onwards (Fig. 4A,B). This could be due to the increase in cell population from the 4th activation onwards where it was observed to increase from 35 × 10^6^ (Day 24) to 75 × 10^6^ (Day 31) and then to 160 × 10^6^ cells (Day 38) leading to the faster depletion of glucose and accumulation of lactate in the media.

The glucose and lactate levels were observed to differ slightly in the Media and cell chamber of BECA-D with the cell chamber having lower glucose and higher lactate level than the media chamber (Fig. 4A,B). This suggests that there may be inefficient media exchange between the chambers in the current design of BECA-D and a potential area for improvement in future versions of the design.

Culture performance is often evaluated by tracking cell population but the process of obtaining a cell count involves sampling a homogenous culture solution which increases handling steps and contamination risks. To determine if glucose levels, which can be obtained easily with a media sample, can be utilized as an inference to cell population, we evaluated the correlation between glucose level and cell number for 24-well plate and BECA-D. A graph of total cell population against total glucose levels for both 24-well plate and BECA-D from 2nd activation to harvest was plotted for each donor (Fig. 4C). An inverse correlation between cell population and glucose level can be observed for the cultures in BECA-D with a Pearson correlation coefficient of − 0.9 suggesting that measurement of glucose levels in the media can potentially be used to accurately determine cell population in BECA-D without the need of a cell count.

## Discussion

In this study, we have optimized the use of BECA-D for the culture of EBVSTs and demonstrated its effectiveness alongside the 24-well plate. We explored three protocols for culture of EBVSTs in BECA-D and confirmed the protocol with culture parameters higher seeding density (1 × 10^6^ cells/cm^2^) and direct seeding at 1st activation into BECA-D achieved the highest yield. In addition, these culture parameters also reduce the complexity of the protocol and minimized handling steps.

In the five donor runs, culture in BECA-D appeared to remain in an exponential phase of growth at the point of harvest (Figs. 2B, 3A) suggesting that there may be potential for the culture to achieve higher numbers through additional activations. However, this strategy of obtaining higher yield compromises on the culture duration resulting in a delay in treatment and higher costs through additional manpower and reagents required. In addition, repeated activations to antigen-specific T cells have been observed to induce tolerance and inhibit their functions^16,17^. To understand the trade-off between yield, culture duration and the quality of EBVSTs obtained, additional studies will have to be conducted to determine the maximum achievable yield for functional EBVST culture in BECA-D.

The study met the aim of matching BECA-D’s performance with 24-well plate across five donors, the results from BECA-D demonstrated statistically, through paired two-tailed t-tests, no significant difference from the 24-well plate in terms of yield, viability, phenotype, and functionality. The high p-value obtained could be due to the low number of replicates performed limited by the number of donor samples and prototypes available for the study. Nevertheless, we observed that the yield in BECA-D exceeded that of the 24-well plate in four donors. The results obtained could be attributed to the design of BECA-D which caters to the sensitivity of T-cells. The expandable culture surface area and feeding strategy of 50% media change through the separate media chamber minimizes manipulations required in the cell chamber and retained a conditioned culture environment within the cell chamber. A conditioned culture environment could be important to T-cell culture as removal of exogenous signals has been observed to be detrimental to T-cells. In addition, the conventional view of lactate as ‘waste’ has been challenged recently with studies showing lactate has observed to be consumed during T-cell activation^18–20^. These studies suggest that the BECA-D setup and feeding strategy confers benefit to the culture by retaining a conditioned culture environment.

Activation of T-cells is known to increase their glucose consumption and insufficient glucose have been known to limit T-cell functionality^21,22^. This phenomenon was also observed in this study, where the 24-well plate culture experienced a drop in glucose level upon 1st activation as measured on Day 10 (Fig. 4A). Decrease in availability of glucose in the media was observed in another study leading to an increased oxygen consumption rate in activated T-cells as the cells switched to oxidative phosphorylation as the mode of ATP generation^23^. In our study, the 24-well plate with the media height of 10 mm might not be able to supply adequate oxygen to the cells to counter the decrease in glucose level, hence adversely affecting the culture. This effect was dampened in BECA-D culture due to the excess media in the media chamber buffering the spike in consumption of glucose and the low media height in the cell chamber supplying adequate oxygen to the media. The glucose and lactate levels were observed to differ slightly in the media and cell chamber of BECA-D (Fig. 4A,B).

The cost and production time involved in the manufacturing T-cells for cell therapy are directly correlated to the manpower available and activation cycles required to achieve the target number of cells. BECA-D was designed to minimize culture handling steps and manpower costs in the manufacturing of cells for cell therapy. Table 2 lists handling steps for culturing EBVST for a well in the 24-well plate and BECA-D for each round of activation. Table 3 lists the total number of handling steps for a hypothetical experiment of a culture that expands 160% after each activation round for five rounds. Over multiple rounds of activations, BECA-D was able to reduce the number of handling steps required by the 24-well plate by more than tenfold, potentially reducing cost and time. The handling of 24-well plate gets exponentially laborious with each round of expansion as the handling steps performed for the 24-well plate had to be repeated for each well. The reduced number of steps in the BECA-D culture is beneficial to the whole culture process as it reduces contamination risks and complexity of the culture process. This translates to easier manpower training, fewer manhours and lower probability of human error during the manufacturing process.

**Table 2:** List of handling steps per process for 24-well plate and BECA-D for expansion of EBVST.

	Handling steps	
	24-well plate	BECA-D
Seeding	Seed PBMCs and LCLs	Add media to media chamber Seed PBMCs and LCLs into cell chamber
Expansion	Collect and pool cells from each well Wash each well Seed cells (T-cells and LCLs) to each well (with or without IL-2)	Collect cells from cell chamber Wash cell chamber Seed cells (T-cells and LCLs) into cell chamber Expand cell chamber Remove 50% of media from media chamber Add fresh media (with or without IL-2) to media chamber
Medium change	Remove 50% of media from each well Add fresh media (with or without IL-2) to each well	Add IL-2 to media chamber
Harvest	Collect and pool cells from each well Wash each well Count cells	Collect cells from cell chamber Wash cell chamber Count cells

The entire culture process is a permutation of the four process—seeding, expansion, medium change and harvest. Handling steps for each process are listed for handling of the 24-well plate and BECA-D.

**Table 3:** Number of handling steps for 24-well plate (24w) and BECA-D for a hypothetical 38-day culture that expands 160% after each activation.

	10^6^ cells	Number of handling steps	
		24w (# wells)	BECA-D
Day 0 Seeding	12	6 (6)	2
Day 10 Expansion	19.2	31 (6 → 19)	6
Day 14 Medium change	19.2	38 (19)	1
Day 17 Expansion	30.7	68 (19 → 30)	6
Day 21 Medium change	30.7	60 (30)	1
Day 24 Expansion	49.3	109 (30 → 49)	6
Day 28 Medium change	49.3	98 (49)	1
Day 31 Expansion	78.6	176 (49 → 78)	6
Day 35 Medium change	78.6	156 (78)	1
Day 38 Harvest	125.8	156 (78)	3
Total		898	33

On expansion day, the number of wells handled for 24-well plate is different before (pooling of cells) and after (reseeding of cells) cell count. The difference is indicated as an arrow e.g. (6 → 19) indicates 6 wells were pooled and 19 wells were seeded.

As previously mentioned in the results section, the protocol used in the study was optimized for the 24-well plate. Switching to larger-well systems could theoretically reduce the manual handling of using culture vessels as the culture expands. However, it has been documented that the yield of antigen-specific T-cells obtained is most optimal when cultured in a 24-well plate and does not perform as well when scaling through larger-well systems^8^. Other than the assumption that antigen-specific T-cells has strict requirements for cell–cell contact thus the importance of culture surface area, it is unclear as to why larger-well systems do not perform as well for these cultures as there have been studies culturing non-antigen-specific T-cells successfully in 6-well plates or T-flasks.

Despite the possibility of scaling through larger-well systems, it maintains to be a non-ideal manufacturing method as the process introduces additional manual steps and open cultureware manipulations—collecting cells from the original plates and seeding to the larger-well plate. In addition, the scaling between different well systems is not linear and adds complexity in considering choice of well system to seed into. These steps are eliminated with the use of BECA which incorporates in-situ expansion of culture area as one of its key features.

Handling steps for BECA-D could be further simplified by omitting the need for cell count, hence omitting sampling for cell count at each round of activation. Cell count is currently required to obtain the cell population number. This is required to calculate the culture area to be expanded for the maintenance of culture density. It is possible to determine cell population number through a surrogate such as glucose level in the media. Glucose is a commonly monitored metabolite in bioprocessing systems and has been used to infer cell numbers in other systems such as CHO cells and hybridomas cultured in packed-bed bioreactors and K562 cells cultured in G-rex^24–26^. In our study, glucose level in the media was observed to be highly correlated (with Pearson correlation coefficient of − 0.9) to cell population in BECA-D suggesting that it has the potential to be a surrogate to determining cell population number omitting the need of a cell count (Fig. 4C). Further work must be performed to test the feasibility of this concept and to develop a model to calculate cell population number from glucose measurements.

We initially included an additional commercially available bioreactor, G-rex10, in the study with Donors 3, 4 and 5. However, EBVSTs cultured in G-rex were unable to achieve yields matched by previous published studies^15,27–29^ (Supplementary Fig. 2, Supplementary Table 1). Culture in G-rex achieved similar phenotypes as the culture in BECA-D and 24-well plate (Supplementary Table 1) but was omitted from functional assays, ELISpot and Cytotoxicity Assay. As we were unable to fully characterize the cultures, the data was omitted in our analysis. The discrepancy in our observations and what was previously documented could have arisen from differences in the culture protocol utilized to expand EBVST. The culture protocol was adapted directly from BECA-D (50% Media Change on days of activation, 1.0 × 10^6^ cells/cm^2^ seeding density) without optimization to G-rex, which might have contributed to the decreased performance in the G-rex. Another possible reason could be starting material variability. It is known that starting material variability often leads to variations in culture performance in different culture vessels and the management of the variability remains a commonly discussed issue within the cell therapy community^30,31^.

Starting material variability could also account for the varying yield of EBVSTs from different donors. A repeated run was performed for Donor 1 and achieved similar yield (data not shown). It is likely that the varying yields observed from Donor 1 to 5 could be attributed to starting material variability.

Starting material variability as well as the issues with application of lab-scale protocols in larger-scale bioreactors highlighted the non-predictability of scaling culture expansion in autologous cell therapy. The availability of a variety of culture vessels with different key features in the market would allow cell therapy developers and manufacturers to better manage the issues during process development for scaling-up and manufacturing.

Looking into current manufacturing needs for Phase III clinical trials involving EBV-specific T-cells, BECA-D is able to satisfy yield requirements for these trials based on results obtained in this study. The dosages indicated in the trials (NCT03392142, NCT04832607, NCT02578641, NCT03394365) range from 2.5 × 10^4^ to 2 × 10^6^ cells/kg which translates to a requirement of 2–160 × 10^6^ cells for an 80 kg patient. These numbers are obtainable from culturing T-cells in 1–3 BECA-Ds. This observation affirms BECA-D’s potential in supporting adoptive cell therapy manufacturing.

This study revealed features of BECA-D that can be improved upon—limitations to volume scaling, inefficient metabolite diffusion across the membrane and need for manual handling for media change and cell/media sampling. Investigations into the redesign of BECA are currently underway to address these issues, including optimizing with cell/media chambers of different sizes, starting and ending plunger positions and integration of automation into these steps. Future iterations of BECA aim to accommodate linear scaling in the manufacturing of a variety of immune cell products with different culture requirements.

Overall, this study has shown that BECA-D was able to achieve EBVST culture performance that matched the 24-well plate using a cost- and time-efficient process. As mentioned above, we are currently developing an automation capsule for BECA-D which would facilitate transfer of a lab-scale process into a closed manufacturing system^9,32^. This would enable stepwise scalability that allows for cell therapy developers to enhance their manufacturing capability with minimal re-optimization to support clinical trials and accelerate entry into market.

## Materials and methods

### Fabrication of bioreactor (BECA-D)

The main body of the bioreactor was made from virgin polystyrene. Parts were injection moulded and assembled via ultrasonic welding. Both the injection moulding and the ultrasonic welding were carried out under an ISO 13485 certified manufacturing environment. The moving plunger was made from overmoulding of polycarbonate core with silicone. The profile, size and hardness of the plunger rubber were optimized to achieve optimal sealing in the cell chamber. The membrane was made from polyethylene terephthalate with a uniform pore size of 1 μm. The membrane was tested with peripheral blood mononuclear cell (PBMC) culture and it was observed cells were unable to cross the membrane via the pores. Chemical diffusion across the membrane was checked with cell culture medium of different glucose concentrations. It was found glucose could slowly diffuse across the membrane from the side of high glucose concentration to the side of low glucose concentration while the culture was kept static. All the materials used for constructing the bioreactor were subjected to in-house biocompatibility test and had shown no adverse effects on the growth of PBMCs.

### Donor recruitment

SingHealth Research Centralised institutional review board approved the proposed human study (CIRB2017/2398) and all methods were performed in accordance with the relevant guidelines and regulations. An email containing the invitation letter and contacts of Principal Investigator was sent to the potential donors (age between 21 and 60 years old, weigh at least 50 kg, generally be in good health) with a request to accept or decline the invitation of blood donation. Written informed consent was obtained from all participants on the day of the blood donation. The donors underwent blood screening to check their eligibility for the blood donation (haemoglobin level of at least 12.5 g/dL). 100 mL whole blood was extracted from eligible donors and processed to obtain Peripheral Blood Mononuclear Cells (PBMCs).

### Cryopreservation of PBMCs

0.1 mL Dimethyl Sulfoxide (DMSO) (ATCC) and 0.9 mL of defibrinated or anticoagulant-treated blood pooled from the same donor was dispensed into each cryovial and mixed periodically to ensure an even cell suspension. The capped vials were transferred to a cryopreservation container in ethyl alcohol. The vials were placed in Mr Frosty Freezing Container (Thermo Fisher Scientific) at − 80 °C for 3–18 h or in a controlled rate freezer of freezing rate of 1 °C per minute prior transferring to storage in liquid nitrogen.

### PBMC preparation

Whole blood was mixed with an equal volume of Phosphate Buffer Saline (PBS) (ATCC) or RPMI-1640 (ATCC) and layered over Histopaque-1077 (Sigma-Aldrich) at 400×*g* for 40 min at room temperature. Once the centrifugation is complete, the opaque lymphocyte band at the intermediate layer and the Histopaque-1077 layer down to within 0.3 cm of the pellet at the bottom of the tube were then transferred to a new centrifuge tube and mixed with Iscove’s Modified Dulbecco’s Medium (IMDM) (ATCC) with 20% Fetal Bovine Serum (FBS) (HyClone Laboratories) and 1% penicillin/streptomycin (ATCC) and centrifuged at 260×*g* for 15 min. The cell pellet was washed once with IMDM with 10% FBS and 1% penicillin/streptomycin and resuspended in IMDM with 10% FBS and 1% penicillin/streptomycin. Cells were immediately used for transformation or cryopreserved for future use.

### Transformation of PBMCs into lymphoblastoid cell lines (LCLs)

Complete culture medium used was IMDM with 10% of FBS and 1% of penicillin/streptomycin solution. A vial of irradiated MRC-5 feeder layer cells (ATCC) was thawed and mixed with complete culture medium. Cell suspension of ~ 4 × 10^5^ cells was dispensed into culture flasks. The medium in the flasks of feeder layers was removed and supplemented with IMDM with 20% FBS, cell suspension containing 1–6 × 10^6^ PBMCs and human gammaherpesvirus 4 (HHV-4) (ATCC). The contents in the flask were gently mixed and incubated at 37 °C under a 5% CO_2_ atmosphere. After 7 days, cells were observed for signs of transformation and complete culture medium was added to the flasks. Every 3–4 days thereafter, cells were observed for transformation and the media was refreshed. Transformed LCL were maintained at the density of 1–3 × 10^5^ cells per mL by expanding to additional flasks. LCLs were then collected from flask and frozen down for future use using chilled freezing medium (complete culture media with 10% DMSO) at the cell density of 3–5 × 10^6^ cells per mL.

### Expansion of EBVST culture

The complete culture medium used for donor PBMC culture was 45% RPMI 1640 (Thermo Fisher Scientific), 45% EHAA Click’s Medium (Irvine Scientific), 10% heat-inactivated FBS (Thermo Fisher Scientific) and 2 mM l-glutamine (Thermo Fisher Scientific).

The complete culture medium used for donor LCL culture was RPMI-1640, 10% heat-inactivated FBS and 2 mM l-glutamine. Cryopreserved LCLs were thawed and maintained at 4 × 10^5^ cells/mL. On days of activation, the required volume of LCLs were removed for irradiation and the remaining cells were passaged and maintained.

### Expansion of EBVST culture (24-well plate)

1st activation (Day 0): PBMCs were thawed and washed to remove DMSO. PBMCs were resuspended in fresh media and cell count was performed and viability determined by Trypan Blue staining (Gibco). PBMCs were seeded into 24-well plate at density of 1.0 × 10^6^ cells/cm^2^. Irradiated LCLs were seeded into the PBMC wells at the ratio of 1:40. Total volume of media in the well was 2 mL. No media change was required until the 2nd activation.

2nd to 5th activation (Day 10, 17, 24, 31): 300 uL of media samples were removed prior to cell collection for glucose and lactate measurements. Cells from each well were pooled and centrifuged at 300×*g* for 10 min at room temperature. Cell pellet was resuspended with complete medium and cell count and viability was determined. Cells were seeded into new 24-well plate at density of 0.5 × 10^6^ cells/cm^2^. Irradiated LCLs were seeded at the ratio of 1:4. 50 IU/mL of Interluekin-2 (IL-2) (STEMCELL Technologies) was added to the culture from 3rd to 5th activation. Total volume of media in the well was 2 mL.

Media Change and IL-2 Top-up (Day 14, 21, 27, 34): 50% media change was performed by removing 1 mL of spent media from the well and replacing it with 1 mL of fresh media. 50 IU/mL of IL-2 was added to each well.

Harvest (Day 38): 300 uL of media samples were removed prior to cell collection for glucose and lactate measurements. Cells from each well were pooled and centrifuged at 300×*g* for 10 min at room temperature. Cell pellet was resuspended with complete medium and cell count and viability were determined. Cells were processed for characterization assays.

### Expansion of EBVST culture (BECA-D)

1st activation (day 0): 180 mL of complete media was added to BECA-D media chamber and 42 mL of complete media was added to BECA-D cell chamber—back. PBMCs were thawed and washed to remove DMSO. Cell pellet was resuspended with complete medium and cell count and viability was determined. PBMCs were seeded into BECA-D cell chamber—front (12 cm^2^) at density of 1.0 × 10^6^ cells/cm^2^. Irradiated LCLs were seeded into the cell chamber at the ratio of 1:40. Total volume of media in the cell chamber was 12 mL. No media change was required until the 2nd activation.

2nd to 5th activation (day 10, 17, 24, 31): 300 µL of media samples were removed from the media chamber and cell chamber—front prior to cell collection for glucose and lactate measurements. Cells from cell chamber were collected, and cell chamber was washed once with complete medium. The medium was pooled with the cells and centrifuged at 300×*g* for 10 min at room temperature. Cell pellet was resuspended with complete medium and cell count and viability was determined. Total surface area of culture was determined using cell number and density of 0.5 × 10^6^ cells/cm^2^ and total volume of culture was determined for a media height of 5 mm. Cells were diluted to appropriate culture volume and seeded into the cell chamber—front. Irradiated LCLs were seeded at the ratio of 1:4 in complete PBMC medium. The Plunger was pulled to the appropriate distance to convey the surface area required for culture. 50% media change was performed by removing 90 mL of spent media from media chamber and replacing it with 90 mL of fresh media. 50 IU/mL of IL-2 was added to the media chamber from 3rd to 5th activation.

IL-2 top-up (day 14, 21, 27, 34): 50 IU/mL of IL-2 was added to the media chamber.

Harvest (day 38): 300 µL of media samples were removed prior to cell collection for glucose and lactate measurements. Cells from cell chamber were collected and cell chamber was washed once with complete medium. The medium was pooled with the cells and centrifuged at 300×*g* for 10 min at room temperature. Cell pellet was resuspended with complete medium and cell count and viability was determined. Cells were processed for characterization assays.

### Cell surface phenotyping

T-cells generated from different culture vessels were transferred into a 1.5 mL Eppendorf tube, centrifuged at 300×*g* for 10 min at room temperature. Cell pellet was resuspended in 100 µL of fluorophore solution and incubated in the dark for 20 min. The samples were washed repeatedly before resuspending in a final volume of 250 µL. MACSQuant Analyser 10 (Miltenyi Biotec) was used to acquire the data and MACSQuantify Software 2.11 (Miltenyi Biotec) was used for analyses. Antibodies used in analyses: Viobility 405/520 Fixable Dye (130-109-814), CD3-VioBlue (130-113-133), CD19-PE (130-113-646), CD56-PE-Vio770 (120-113-313), CD4-APC-Vio770 (130-113-251) and CD8-FITC (130-110-677) (Miltenyi Biotec).

### Enzyme-linked immunospot (ELISpot)

Human interferon gamma (IFN-ʏ) ELISpot Plus Kit (3420-4 HST-10) (Mabtech) was used to quantify IFN-ʏ secreting T-cells in vitro. A human IFN-ʏ pre-coated strip plate was prepared according to manufacturer’s protocol. 20 µL of 6.25 μg/mL antigens or controls and 100 µL of 2.5 × 10^6^ cells/mL T-cells were added to each well. The antigens used were LMP1 PepMix, LMP2 PepMix, EBNA1 PepMix. DMSO was used as a negative control and CD3 mAb 6 μg/mL was used as a positive control. The plate was analysed using the C.T.L ImmunoSpot Analyzer Software 5.2 (Cellular Technology Limited (CTL)).

### Cytotoxicity assay

Cytotoxicity capabilities of the cultures were assessed with the DELFIA^®^ EuTDA Cytotoxicity Reagents (PerkinElmer) using Effector:Target (E:T) ratios ranging from 40:1 to 2.5:1, with matched LCLs as the target. The cytotoxicity assay was performed in a 96-well plate accordance to the manufacturer’s protocol. The plate was assessed with a Tecan Infinite 200 PRO (Tecan). Specific killing was calculated as follows:

$$\small \%\ Specific\, killing=\frac{(Experimental \,release\, \left(counts\right)-Spontaneous\, release\, \left(counts\right))}{(Maximum\, release\, \left(counts\right)-Spontaneous\, release\, \left(counts\right))}\,\times 100\%$$

where labelled LCLs incubated in medium alone or in 2% Triton x-100 (Sigma-Aldrich) were used to determine Spontaneous and Maximum release respectively.

### Glucose and lactate measurements

Frozen media were thawed and analysed for glucose and lactate levels using the Cedex Bio Analyzer (Roche Diagnostics). Glucose and lactate concentrations were measured accordance to the manufacturer’s protocol.

### Statistical analysis

Paired Student’s two-tailed t-test was performed to assess the statistical significance of differences between two groups across the donor sets. A p-value of < 0.05 obtained from the test indicates a significant difference. Correlation between glucose media level and cell numbers were evaluated using Pearson’s correlation coefficient.

## Supplementary Information

Supplementary Information.
