# Supplementary material for: In-situ scalable manufacturing of Epstein–Barr virus-specific T-cells using bioreactor with an expandable culture area (BECA)
Source: Sci Rep. 2022 Apr 29;12:7045. doi: 10.1038/s41598-022-11015-z (PMC9054749; doi:10.1038/s41598-022-11015-z)
Supplement: Supplementary file 1 — Supplementary Information. [file 41598_2022_11015_MOESM1_ESM.pdf]

In-situ scalable manufacturing of Epstein-Barr Virus-Specific T-cells using Bioreactor with an Expandable Culture Area (BECA)

Sixun Chen[1], Ahmad Amirul Bin Abdul Rahim[1], Who-Whong Wang[2], Rachael Cheong[2], Akshaya V Prabhu[1], Jerome Zu Yao Tan[1], May Win Naing[3], Han Chong Toh[2], Dan Liu[1]*

[1]Biomanufacturing Technology, Bioprocessing Technology Institute, A*STAR, 20 Biopolis Way, Singapore 138668, Singapore

[2]Division of Medical Oncology, National Cancer Centre Singapore, 11 Hospital Cres, Singapore 169610, Singapore

[3]Singapore Institute of Manufacturing Technology (SIMTech), A*STAR, 2 Fusionopolis Way, Singapore 138634, Singapore

***liu_dan@bti.a-star.edu.sg**

Supplementary Figures

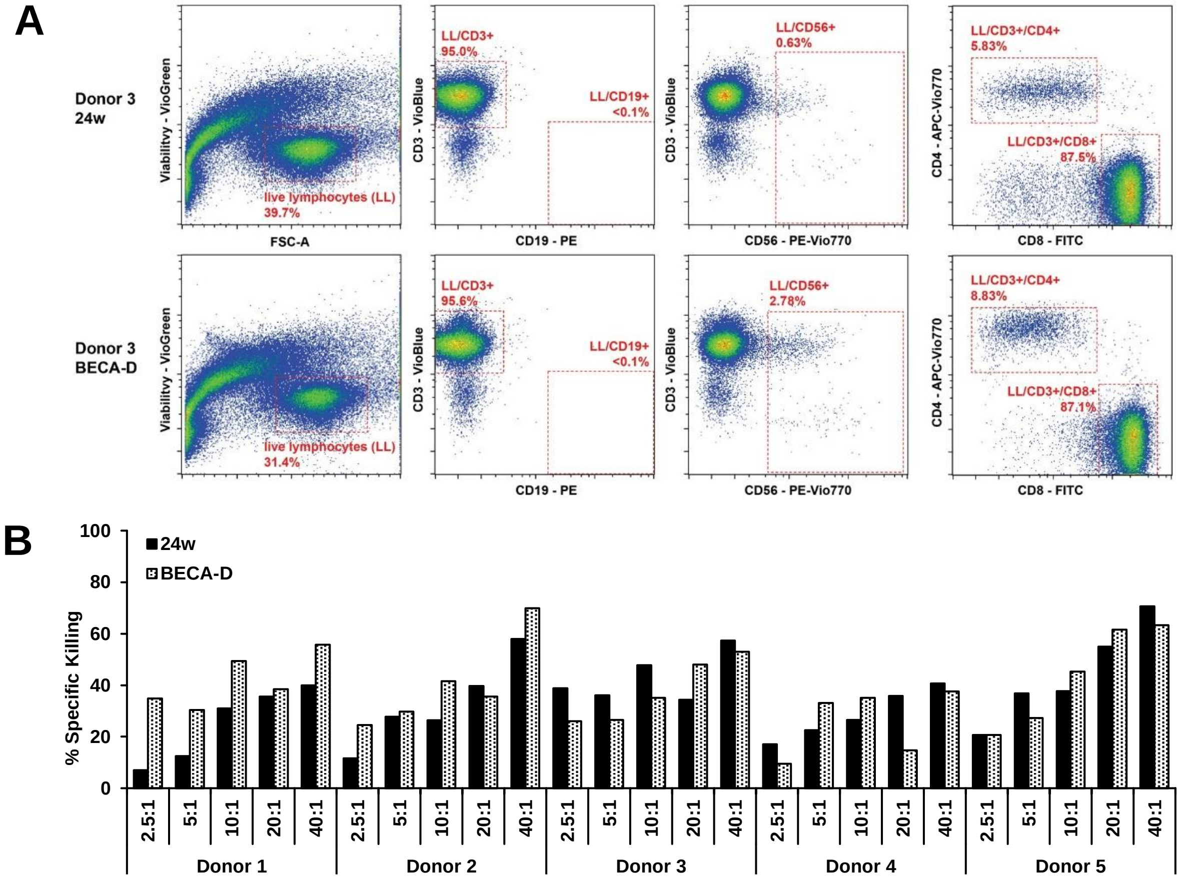

Supplementary Figure 1. (A) Representative flow cytometry plots showing gating strategy of CD3+ T-cells, CD19+ B-cells, CD56+ NK cells, CD4+ helper T-cells and CD8+ cytotoxic T-cells. (B) Performance of 24w and BECA-D culture across five donors in cytotoxicity assay with LCL:CTL ratios as indicated.

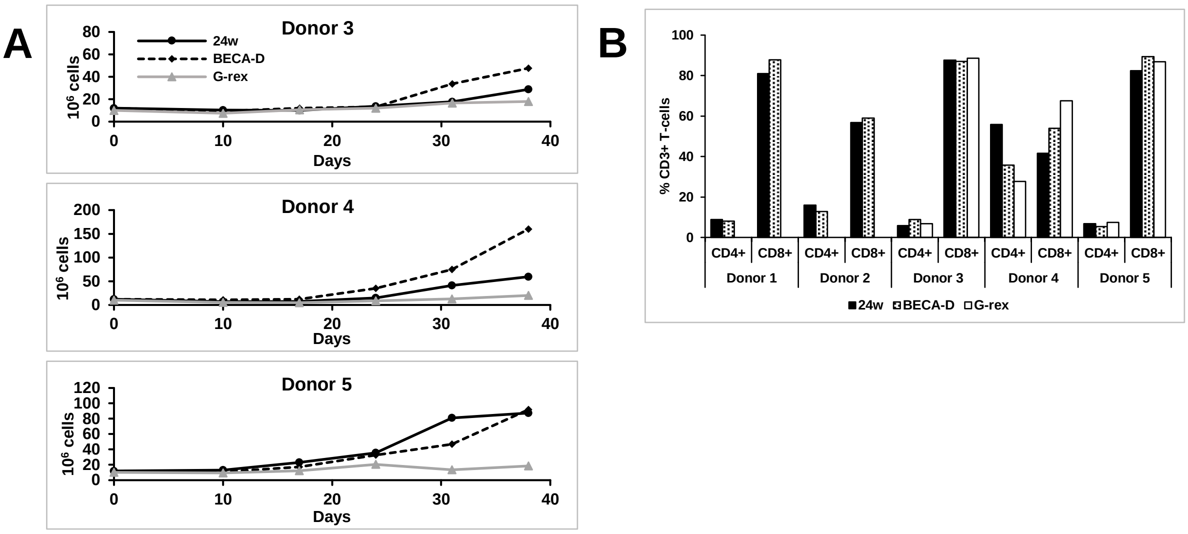

Supplementary Figure 2. Expansion of EBVST in 24-well plate (24w), BECA-D and G-rex. (A) Plot of cell population numbers counted for 24-well plate (24w), BECA-D and G-rex at the end of every activation for Donor 3, Donor 4 and Donor 5. (B) Percentage population of helper (CD4+) and cytotoxic (CD8+) T-cells under the subset of T-cells (CD3+) cells at the end of 5th activation for Donors 1-5. G-rex culture was performed on Donor 3, 4 and 5.

	Vessel	*Donor 1*	*Donor 2*	*Donor 3*	*Donor 4*	*Donor 5*
**Yield / Viability**						
Cell population 10^6 cells (% Viability)	24w	10.3 (90%)	81.6 (78%)	28.8 (69%)	59.5 (85%)	87.4 (94%)
	BECA-D	129.1 (95%)	79.1 (91%)	47.7 (81%)	160.0 (90%)	91.9 (91%)
	G-rex	-	-	18.0 (69%)	20.0 (85%)	18.5 (94%)
**Phenotype**						
% CD3	24w	93.5	96.8	95.0	95.1	93.7
	BECA-D	96.9	95.4	95.6	96.0	93.0
	G-rex	-	-	90.7	94.8	91.2
% CD19	24w	<0.1	<0.1	<0.1	<0.1	<0.1
	BECA-D	<0.1	<0.1	<0.1	<0.1	<0.1
	G-rex	-	-	<0.1	<0.1	<0.1
% CD56	24w	20.6	1.5	0.6	0.5	0.6
	BECA-D	7.6	5.3	2.8	3.1	0.3
	G-rex	-	-	0.7	1.0	0.4
% CD3/CD4	24w	8.8	15.9	5.8	55.8	6.8
	BECA-D	8.1	12.8	8.8	35.7	5.4
	G-rex	-	-	6.8	27.7	7.5
% CD3/CD8	24w	81.0	56.7	87.6	41.6	82.4
	BECA-D	87.8	59.0	87.1	53.9	89.4
	G-rex	-	-	88.6	67.5	86.9

Supplementary Table 1: Results of EBV-CTL expansion process in 24-well plate, BECA-D and G-rex. Summary of results (yield, viability and phenotype) of the culture in 24-well plate, BECA-D and G-rex from five different donor runs.
